# Non-Resolving Repair in Idiopathic Pulmonary Fibrosis: From Failed Cellular Transitions to Architectural Lock-In

**DOI:** 10.3390/ijms27177649

**Published:** 2026-08-26

**Authors:** Chuang Ge, Chaoyue Cui

**Affiliations:** Center for Biological Science and Technology, Key Laboratory of Cell Proliferation and Regulation Biology, Ministry of Education, Guangdong Zhuhai-Macao Joint Biotech Laboratory, Department of Biology, Faculty of Arts and Sciences, Beijing Normal University, Zhuhai 519087, China; 202531079069@mail.bnu.edu.cn

**Keywords:** idiopathic pulmonary fibrosis, non-resolving repair, spatial omics, single-cell transcriptomics, fibrotic niche, resolution capacity, lesion unit

## Abstract

Repair programs are pervasive in idiopathic pulmonary fibrosis (IPF), yet they fail to reach completion. While single-cell and spatial omics have identified cellular states associated with injury-repair programs, a central paradox remains: why do these programs persist without reconstituting functional lung architecture? This review integrates evidence from multiscale omics, spatial analyses, and translational studies to propose a lesion-centered “non-resolving repair” framework that explains IPF progression. We argue that disease progression is driven by the persistence of cellular repair programs after the pathways required for maturation, state exit, and microenvironmental reset have become compromised. These compartment-specific failures converge within spatially organized lesion units, where aberrant cellular activity and matrix distortion reinforce one another, embedding failed repair within tissue architecture. This perspective shifts the focus from cataloging disease-associated cell states toward evaluating failed biological transitions and regional resolution capacity. Clinically, it reframes antifibrotic therapy around overcoming spatial barriers to repair and highlights the need for translational endpoints that distinguish marker suppression from structural stabilization and functional tissue reconstruction. Delineating which lesional niches retain resolution capacity will be essential for identifying where repair-oriented interventions may still re-engage organized tissue repair in IPF.

## 1. Introduction

Idiopathic pulmonary fibrosis (IPF) remains a disease in which clinical therapeutic advances have outpaced our mechanistic understanding of tissue repair. While current antifibrotic therapies slow functional decline, established IPF inevitably progresses to the irreversible loss of distal lung architecture and respiratory failure. The inability to reverse disease progression highlights a fundamental biological failure: following injury-triggered remodeling, the fibrotic lung cannot re-establish a functional alveolar architecture [1,2].

Fibrosis has long been understood as an outcome of dysregulated wound repair. While extracellular matrix (ECM) deposition initially serves as a protective response to stabilize injured tissue, it drives pathological matrix accumulation and architectural distortion when poorly coordinated. In IPF, this dysregulation is continuously fueled by the interplay of repetitive epithelial injury, aging, immune dysfunction, and sustained fibroblast activation. The critical biological question, therefore, is why these injury-response programs fail to regress and permit architectural restoration [3,4,5].

Recent advances in single-cell and spatial omics have provided unprecedented resolution to this problem, mapping diverse epithelial, stromal, immune, and vascular states organized within specific anatomical niches. However, interpreting these high-resolution maps requires a conceptual shift: an injury-induced cell state is not intrinsically pathological. It drives disease only when it persists, loses its developmental exit route, or becomes trapped in a fibrotic niche that precludes structural resolution [6,7,8].

To bridge this conceptual gap, this review introduces non-resolving repair as an organizing framework. We define non-resolving repair as a state in which injury-response and repair programs remain active after the temporal, cellular and architectural conditions required for closure have deteriorated. The concept emphasizes failed transitions: epithelial plasticity without mature alveolar reconstruction, fibroblast activation without withdrawal, immune cleanup without disengagement, vascular remodeling without rebuilding of the alveolar–capillary interface, and matrix remodeling without restoration of tissue geometry. Crucially, in progressive IPF, they become spatially and mechanistically coupled within local lesion niches.

Consequently, this framework shifts the analytical scale of IPF progression: the pathogenic potential of an aberrant cell state depends intrinsically on its duration, anatomical niche, and surrounding matrix architecture. The relevant unit of disease progression is therefore not an isolated cell population, but an integrated lesion unit where epithelial, stromal, immune, and vascular programs mutually reinforce one another. Once established, these microenvironmental fields progressively reshape adjacent tissues, altering the baseline for subsequent repair and effectively precluding the return to a normal alveolar template.

**Figure 1:**
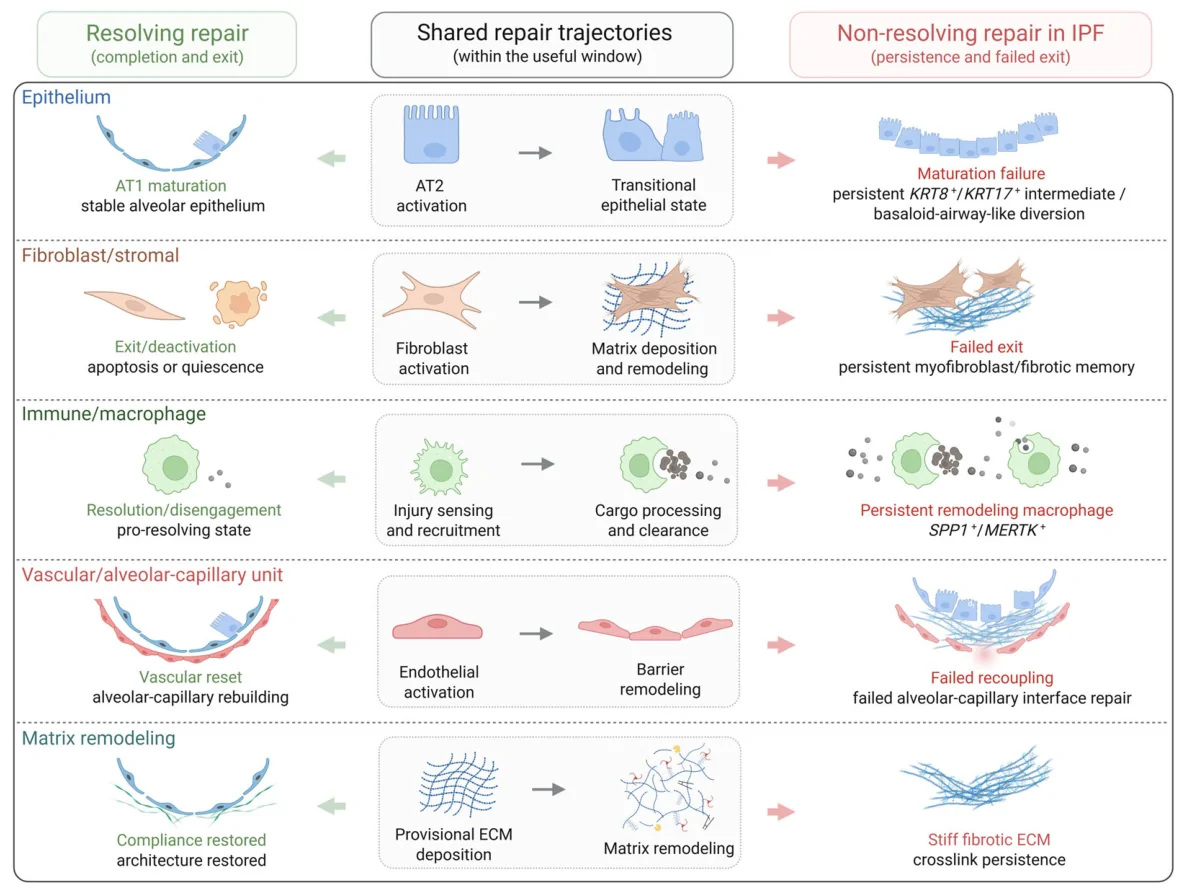
Shared repair trajectories diverge toward repair completion or non-resolving repair in IPF. The epithelial, fibroblast/stromal, immune-macrophage, vascular/alveolar–capillary, and matrix-remodeling programs activated after injury are not intrinsically pathological. At early stages, these programs jointly contribute to damage containment, temporary tissue support, clearance of injury-associated cargo, and restoration of barrier integrity. When each module completes its task and exits within an appropriate “useful window,” repair proceeds toward AT1 epithelial maturation, fibroblast deactivation or clearance, immune resolution, rebuilding of the alveolar–capillary unit, and restoration of compliant matrix architecture. By contrast, in IPF, these normally beneficial repair programs persist and become diverted: epithelial intermediates fail to mature and shift toward *KRT8*^+^/*KRT17*^+^ or basaloid-airway-like aberrant states; fibroblasts remain in matrix-producing myofibroblast states; remodeling macrophages persist together with unresolved cargo; vascular repair fails to re-establish the epithelial-capillary interface; and provisional matrix gradually evolves into stiff, crosslinked fibrotic ECM. This framework emphasizes that the central problem in IPF is the failure of repair programs to complete, exit, and recouple into a functional gas-exchange architecture. Abbreviations: AT1, alveolar type 1 epithelial cell; AT2, alveolar type 2 epithelial cell; ECM, extracellular matrix; IPF, idiopathic pulmonary fibrosis. Created in BioRender. Ge, C. (2026) https://BioRender.com/cwtsice.

This review explores the non-resolving repair framework across multiple biological scales, first detailing how epithelial, fibroblastic, immune, and vascular repair programs become persistently arrested. We next delineate how these arrested programs converge into discrete lesion units that propagate through the lung, driving spatial and architectural lock-in. Finally, we discuss strategies to map the tissue’s intrinsic “resolution capacity” and propose reframing therapeutic development around overcoming these specific barriers to repair. By shifting the focus beyond isolated cellular catalogs, we aim to uncover whether and how fibrotic lung tissue can re-engage organized and functional regeneration. This multiscale framework is summarized in Figure 1.

## 2. Arrested Epithelial Repair: Transitional States at the Boundary of Regeneration and Remodeling

The distal lung epithelium has long been central to IPF pathogenesis. Classical models emphasized epithelial depletion, alveolar type 2 (AT2) cell apoptosis, and barrier dysfunction. High-resolution omics have expanded this view by revealing an active and highly plastic epithelial landscape. IPF lungs contain aberrant states that do not align cleanly with normal alveolar type 1 (AT1), AT2, or airway lineages and instead combine features of injury response, aborted regeneration, cellular stress, and airway-like remodeling [6,9]. These findings frame epithelial pathology as unresolved repair, in which the epithelium remains trapped between regeneration and fibrotic remodeling.

### 2.1. Transient Epithelial Plasticity as a Normal Route of Alveolar Repair

Alveolar epithelial repair necessitates a temporary destabilization of lineage identity. Following injury, AT2 cells downregulate their steady-state surfactant programs to proliferate, enter a transitional state, and ultimately differentiate into AT1 cells to restore the gas-exchange surface. Crucially, this regenerative plasticity functions as a physiological repair mechanism provided that stress-induced transcriptional programs remain reversible, allowing the intermediate cell to successfully exit the transitional state.

Recent experimental models have characterized several such transitional populations. During regeneration, differentiating AT2 cells transit through a pre-alveolar type 1 transitional cell state (PATS) [10]. Similarly, a *Krt8*^+^ alveolar differentiation intermediate (ADI) emerges across various lung injury models, coupling epithelial repair to p53, nuclear factor kappa B (NF-κB), and senescence-associated pathways [11]. Additionally, inflammatory cues like interleukin-1 beta (IL-1β) can prime AT2-lineage cells into damage-associated transient progenitors (DATPs) through a hypoxia-inducible factor 1 alpha (HIF-1α)-dependent glycolytic program [12]. Despite arising from disparate experimental contexts, these diverse states underscore that AT2-to-AT1 differentiation is a staged process requiring a transient intermediate phase.

Thus, keratin expression, inflammatory signaling, stress-response genes and senescence-like features are insufficient on their own to define a transitional epithelial state as pathogenic. Many of these features are part of the transcriptional and functional burden of repair. The critical pathogenic divergence in IPF is the failure to close this transitional window, resulting either in stalled AT1 maturation or the redirection of epithelial plasticity toward abnormal identities that cannot reconstruct a functional alveolus.

### 2.2. Persistence and Diversion of Transitional Epithelial States in IPF

In IPF, AT2-to-AT1 differentiation stalls, characterized by the accumulation of *KRT8*-high transitional cells that are unable to reconstruct a mature gas-exchange surface [13]. Single-cell transcriptomics reveals that this stall frequently couples with lineage diversion, giving rise to aberrant basaloid cells near myofibroblast foci (co-expressing basal, mesenchymal, and senescence markers) and *KRT17*^+^ pathological populations with ECM-associated signatures [8,14]. The epithelial repair program deviates from alveolar restoration toward a pro-fibrotic phenotype.

Unlike in murine models where transitional states resolve alongside injury, these epithelial intermediates persist in human IPF, progressing toward aberrant basaloid phenotypes driven by epithelial-stromal and immune feedback [15].

### 2.3. Airway-like and Basal-like Diversion of Distal Epithelial Repair

In severely remodeled distal lung regions, epithelial programs frequently undergo airway-like or basal-like diversion, repopulating damaged alveoli with programs resembling airway repair (e.g., basal cell expansion, *KRT17*-high dedifferentiation, bronchiolization, and honeycomb cyst formation) rather than a mature AT1/AT2 surface. Hedgehog activation in *Gli1*^+^ mesenchymal stromal cells contributes to this diversion by enhancing BMP antagonism and directing airway progenitors toward KRT5^+^ metaplasia during fibrotic repair [16]. At the cellular and anatomical levels, IPF-derived airway basal cells exhibit a profibrotic *KRT17*-high/*PTEN*-low phenotype, while GPR87^+^ basal-like cells are highly enriched within distal honeycomb cysts, structurally anchoring this lineage diversion to end-stage remodeling [17,18]. Ultimately, this lineage misdirection allows airway-like programs to occupy and stabilize abnormal fibrotic architecture, precluding functional alveolar regeneration.

### 2.4. Regulatory Constraints on Epithelial Maturation

Epithelial maturation requires precise spatiotemporal coordination of inflammation, lineage identity, mechanical signaling, and cellular stress; in IPF, the dysregulation of these controls traps cells in transitional states. For instance, while early inflammatory cues like IL-1β prime AT2 cells into regenerative DATPs, their chronic persistence in fibrotic microenvironments prevents AT1 maturation, locking the epithelium in stress-associated intermediate states [12]. Intrinsic lineage-stabilizing machinery is equally critical, as evidenced by CEBPA, which normally restricts AT2 cell plasticity during repair; its repression disrupts AT2 identity and drives pulmonary fibrosis, hindering the intrinsic recovery of an alveolar program [19,20].

Mechanotransduction via the Hippo pathway further exemplifies this temporal dependency: transient activation of yes-associated protein (YAP) and transcriptional coactivator with PDZ-binding motif (TAZ) in AT2 cells is essential for AT1 regeneration, whereas sustained YAP/TAZ activity promotes aberrant differentiation and persistent fibrotic remodeling [21,22]. Finally, proteostatic stress (e.g., ER stress and altered surfactant processing) and telomere dysfunction-induced senescence further exhaust the regenerative capacity of AT2-lineage cells, effectively blocking the route from injury adaptation to mature alveolar differentiation [23,24,25].

### 2.5. Spatial Placement of Arrested Epithelial States at the Repair–Remodeling Interface

Spatial omics reveal that aberrant epithelial states colocalize within transforming growth factor beta (TGF-β)-enriched fibrotic niches, placing them at the repair–remodeling interface [26,27]. Their spatial placement suggests that persistent epithelial intermediates participate in local repair–remodeling interactions. Functional studies further show that selected aberrant intermediate populations can promote fibroblast activation [28], and depleting *Sprr1a*-positive intermediates directly attenuates bleomycin-induced pulmonary fibrosis [29].

Taken together, these spatial and functional findings redefine the epithelial pathology of IPF. The diverse intermediate states, such as *KRT8*-high cells, DATPs, and aberrant basaloid cells, occupy distinct positions along or adjacent to the alveolar repair trajectory, exhibiting varying degrees of maturation potential, pathological retention, and lineage diversion. Epithelial non-resolution is thus characterized by a loss of regenerative trajectory: restricted by regulatory constraints and anchored within fibrotic niches, the epithelium fails to reconstruct the gas-exchange surface, instead functioning as a persistent driver of stromal activation.

## 3. Pathological Fibroblast Repair: Matrix-Producing States That Fail to Exit

During physiological wound repair, fibroblast activation, characterized by expansion, contractility, and matrix deposition, is a transient response that stabilizes damaged tissue and subsequently withdraws. In IPF, this withdrawal phase fails; matrix-producing fibroblasts persist within fibroblastic foci, driving continuous collagen accumulation and dense scar formation.

### 3.1. From Wound-Stabilizing Activation to Persistent Scar-Building

Recent single-cell and spatial atlases have redefined the fibrotic mesenchyme, revealing that scar formation is driven by diverse stromal programs rather than a single canonical myofibroblast population [30]. For instance, a highly fibrogenic *CTHRC1*^+^ fibroblast subset uniquely emerges within fibrotic lesions, directly linking a specific transcriptional state to intense collagen production and scar architecture [31].

These *CTHRC1*^+^ cells conceptually overlap with *POSTN*^+^, *FAP*^+^, and *ACTA2*^+^ matrix-remodeling states, underscoring that the fibrotic mesenchyme reorganizes into a network of distinct but functionally cooperative subsets committed to sustained extracellular matrix production [32].

Crucially, these pathological states arise from the dysregulation of diverse, pre-existing tissue-resident lineages (such as alveolar fibroblasts) rather than the simple expansion of a preformed fibrotic cell type [33]. Thus, the stromal pathology in IPF represents a collective failure of normal mesenchymal populations to exit their activated, wound-stabilizing states.

### 3.2. Apoptotic Exit Failure

Apoptosis represents one major route of myofibroblast exit following normal wound stabilization. In IPF, the failure to eliminate these matrix-producing cells prevents fibrosis resolution regardless of active matrix degradation or inflammatory shutdown.

The Fas-mediated apoptotic gate is frequently disabled in fibrosis-associated fibroblasts through multiple distinct mechanisms [34]. Specifically, molecules such as cellular FLICE-like inhibitory protein and protein tyrosine phosphatase N13 actively divert Fas signaling from death toward survival and proliferation [35,36]. Furthermore, the loss of Thy-1 disrupts Fas localization in lipid rafts, shielding these cells from apoptotic clearance and ensuring their persistence within the scar [37].

Upregulation of anti-apoptotic BCL-2 proteins provides another major survival advantage. Genetic overexpression of BCL-2 in fibroblasts prevents their normal clearance and drives durable scarring. Conversely, targeted inhibition with agents like ABT-263 successfully re-engages the apoptotic program to resolve fibrotic remodeling, demonstrating that the failure of this death exit helps maintain the fibrotic architecture [38,39].

### 3.3. State Exit Failure: Deactivation and Phenotype Reversal

Beyond apoptotic clearance, fibroblasts can achieve resolution through active state withdrawal, characterized by the downregulation of contractile programs and a phenotypic shift toward lipogenic or alveolar-supportive identities. Lineage-tracing studies confirm this plasticity by demonstrating that myofibroblasts can revert to a lipofibroblast-like phenotype during fibrosis resolution [40], refuting the concept of myofibroblast differentiation as a strictly terminal fate.

Pharmacological interventions can actively exploit this state exit. Metformin, for example, accelerates fibrosis reversal by inducing lipogenic differentiation in myofibroblasts [41]. Recent single-cell analyses further map this reversible switch, detailing the bidirectional transition between lipofibroblast-like states and pathogenic *Cthrc1*-positive myofibroblasts [42].

In IPF, however, this stromal state plasticity is progressively lost. Chronic niche cues, including recurrent epithelial stress, immune remodeling, and matrix stiffness, repeatedly reinforce matrix-producing programs. This persistent signaling renders diverse stromal lineages increasingly resistant to deactivation, effectively impeding the phenotypic withdrawal required for tissue resolution.

### 3.4. Fibrotic Memory: Why Fibroblasts Do Not Reset Cleanly

Even upon the cessation of initiating injuries, fibrosis-associated fibroblasts often fail to reset due to acquired mechanical and epigenetic memory, rendering the scar-building program intrinsically resistant to deactivation. At the mechanical level, a rigid extracellular matrix establishes a self-sustaining feedback loop via YAP/TAZ signaling, wherein the deposited scar continually reinforces cytoskeletal tension and further collagen production [43]. Concurrently, chromatin remodeling driven by transcription factors such as TWIST1, AP-1, EGR, and RUNX1 can establish a persistent regulatory memory within the mesenchymal compartment [44,45,46]. This epigenetic memory confers amplified profibrotic hyper-responsiveness to recurrent epithelial injuries, structurally locking fibroblasts in an active state long after the initial insult has passed.

### 3.5. Spatial Anchoring in Fibroblastic Foci and Dense Scars

Spatial transcriptomics reveals that fibrosis-associated fibroblasts expressing *CTHRC1*, *FAP*, and *POSTN* concentrate specifically within subepithelial regions beneath extensive epithelial metaplasia. This precise anatomical localization structurally anchors scar-building stromal programs directly at the interface of aberrant epithelial remodeling [27]. Moreover, *WNT5A*/*CTHRC1*-positive and PAK2-activated myofibroblast states populate both fibroblastic foci and dense fibrosis [47,48], indicating that stromal activity persists within dense scar tissue.

Taken together, these multi-dimensional findings redefine the stromal pathology of IPF. The diverse matrix-producing populations represent a collective failure of the mesenchyme to exit its wound-stabilizing phase. Fibroblast non-resolution is thus characterized by a progressive loss of withdrawal capacity: sustained by apoptosis resistance, persistent phenotypic activation, and acquired fibrotic memory, these cells ultimately become structurally locked within the dense scar architecture. The stromal compartment fails to undergo physiological deactivation, transforming instead into a persistent, self-maintaining fibrotic barrier.

## 4. Incomplete Immune Resolution: Clearance Programs Redirected Toward Remodeling

The failure of broad immunosuppression in IPF indicates that the fibrotic immune landscape extends beyond classical inflammatory excess [49]. Instead, immune cells actively execute fundamental injury-response tasks—sensing danger, clearing debris, and coordinating stromal repair. However, in IPF, this physiological cleanup program becomes perpetually arrested, leaving immune cells trapped in a continuous cycle of injury processing without achieving tissue restoration.

### 4.1. Immune Resolution as an Active Repair Transition

Physiological immune resolution requires an actively organized transition, often driven by specialized pro-resolving mediators (SPMs), to efficiently clear apoptotic debris and subsequently contract macrophage responses [50]. In IPF, however, this active transition is derailed. The persistent stress from aberrant epithelium, disorganized matrix, and vascular dysfunction continuously supplies injury cues, trapping recruited monocytes and macrophages in an unremitting clearance phase. Single-cell and spatial omics validate this derailment by localizing distinct immune states, such as *SPP1*/*MERTK*^+^ macrophages, specifically within fibrotic niches [7,51]. Rather than disengaging to allow tissue quiescence, these immune cells remain structurally entrenched, directly coupling their cleanup functions to ongoing stromal remodeling.

### 4.2. Efferocytosis Failure and Misdirected Clearance

Efficient efferocytosis of dying cells is essential to prevent secondary necrosis and shift macrophages toward inflammatory resolution; however, IPF alveolar macrophages exhibit intrinsically reduced efferocytic capacity, leading to the pathological accumulation of apoptotic cells and a persistent danger-rich environment [52]. Beyond impaired clearance, the continuous influx of apoptotic alveolar epithelial cells actively corrupts macrophage function, as experimental models demonstrate that engulfing this specific pathological cargo is sufficient to trigger profibrotic macrophage activation and initiate fibrosis [53]. Thus, efferocytosis in IPF is compromised on two interacting fronts: intrinsic phagocytic defects allow damage-associated signals to persist, while the chronic processing of apoptotic epithelial cargo continually programs macrophages toward fibrotic remodeling rather than resolution.

### 4.3. Matrix Clearance Versus Matrix Remodeling

Beyond apoptotic cells, macrophages must process extracellular matrix fragments and tissue debris, which promotes resolution only if successful clearance allows tissue architecture to normalize. In IPF, however, macrophages fail to accomplish this clearance, remaining in prolonged contact with the remodeling scar. For example, while ApoE/LRP1-dependent collagen phagocytosis by monocyte-derived macrophages drives fibrosis resolution in bleomycin-induced models, such matrix-engaging programs in IPF become maladaptive [54].

Rather than culminating in post-clearance contraction, IPF macrophages remain trapped within collagen-rich, fibroblast-dense niches. Consequently, continuous exposure to stromal and epithelial remodeling cues hijacks their matrix-processing machinery, actively converting a scar-clearance program into a persistent driver of scar remodeling.

### 4.4. Monocyte-Derived Macrophage Persistence

Instead of retreating after the acute injury phase, recruited monocyte-derived alveolar macrophages (Mo-AMs) often become durable residents of the remodeled lung. Their persistent pathogenic role is highlighted by experimental models where the targeted removal of these recruited macrophages successfully attenuates lung fibrosis [55]. This persistence is actively maintained by local niche signals. Macrophage colony-stimulating factor (M-CSF) and its receptor (M-CSFR) sustain Mo-AMs within spatially restricted fibrotic regions, anchoring them near fibroblasts and abnormal matrix to create a mutually reinforcing loop of immune processing and stromal remodeling [56].

Clinical observations across fibrotic lung diseases corroborate this mechanism. Notably, in patients with post-COVID-19 pulmonary fibrosis, the expansion and CCL2-associated recruitment of profibrotic Mo-AMs directly correlate with radiographic disease severity, demonstrating a conserved paradigm where recruited macrophages fail to transition into reparative states once acute injury subsides [57].

### 4.5. Interpreting Injury-Response Macrophage Programs

Although persistent macrophage programs in IPF characteristically express genes such as *SPP1*, *GPNMB*, *APOE*, and *TREM2*, comparative models demonstrate that these markers define a conserved response to tissue damage rather than a fibrosis-specific lineage. Specifically, *Gpnmb*- and *Spp1*-expressing recruited macrophages populate self-resolving lung injuries during normal tissue repair, indicating that such transcriptional signatures reflect a general physiological adaptation to injury [51,58]. Therefore, static marker expression blurs the boundary between transient, repair-associated activation and progressive, remodeling-associated persistence. The pathological significance of these macrophage states cannot be inferred from transcriptional identity alone; rather, their interpretation critically depends on whether they dynamically resolve or remain spatially sequestered within active fibrotic niches.

### 4.6. Pro-Resolving Programs and Therapeutic Re-Engagement

Therapeutic targeting of SPMs underscores that immune resolution is an actively instructed process. Because SPMs coordinate inflammatory shutdown and debris clearance without broad immunosuppression, they provide a potential route for therapeutically re-engaging immune resolution [50]. Experimental models validate that this resolution program remains accessible for intervention, as both the systemic administration of 17(R)-resolvin D1 and the targeted pulmonary delivery of SPM-based nanotherapeutics successfully attenuate lung fibrosis by restoring these impaired clearance pathways [59,60].

Overall, the immune pathology of IPF reflects a physiological cleanup program persistently derailed by its microenvironment. Trapped within niches of continuous epithelial stress and aberrant matrix, macrophages relentlessly process pathological cargo without ever achieving post-clearance contraction. This unremitting engagement essentially hijacks the very cells designated to clear the scar, integrating them directly into the self-sustaining machinery that preserves it.

## 5. Failed Alveolar–Capillary Repair: Vascular Dysregulation and Defective Gas-Exchange Unit Reconstruction

True tissue resolution culminates in a final structural imperative: the reconstruction of a functional gas-exchange unit. This requires the precise spatial re-alignment of a thin AT1 epithelial surface, a specialized alveolar capillary endothelium, and a minimal interstitial matrix. In IPF, even if disparate fibrotic elements are altered, they fail to spontaneously reassemble into this highly coordinated interface, defining the ultimate structural endpoint of non-resolving repair.

### 5.1. The Endpoint of Repair Is a Gas-Exchange Unit

Because AT1 cells cannot support gas exchange in isolation, successful alveolar repair demands highly specific capillary reconstruction rather than generic angiogenesis. Specifically, it relies on the re-establishment of a distinctly specialized capillary bed comprising aerocyte capillary endothelial cells (aCap cells) specialized for gas exchange and general capillary endothelial cells (gCap cells) involved in homeostatic regulation [61].

Recent comprehensive single-cell atlases of the human lung vasculature emphasize this intricate phenotypic diversity, underscoring that failed repair cannot be judged by the persistence of fibrotic markers alone [62,63]. The definitive endpoint of tissue restoration necessitates the precise anatomical realignment of this delicate epithelial–capillary interface, rather than replacing damaged parenchyma with a haphazardly vascularized scar.

### 5.2. Endothelial and Vascular States in Fibrotic Lung

Vascular remodeling in IPF is characterized by the simultaneous loss of alveolar capillary identity and the emergence of aberrant vascular programs. Single-cell transcriptomic analyses corroborate this shift, identifying the ectopic expansion of a COL15A1^+^CD31^+^ endothelial population that phenotypically resembles bronchial-restricted endothelium within the fibrotic distal lung [8]. Fibrotic vascular pathology therefore arises from a fundamental misdirection of endothelial identity and anatomical placement, extending well beyond simple capillary rarefaction.

This proangiogenic, airway-like endothelial displacement represents a conserved mechanism of parenchymal remodeling across diseases. Indeed, COL15A1^+^ vessels similarly mark ectopic vasculature expansion in lung adenocarcinoma [64] and directly replace normal alveolar capillaries during tissue remodeling in nonspecific interstitial pneumonia (NSIP) [65].

Concurrently, fibrotic microenvironments lock venous and capillary endothelium into persistently activated, injury-responsive states. In aging-associated progressive fibrosis models, these compartments undergo metabolic and transcriptional reprogramming enriched for hypoxia, glycolysis, and sustained YAP/TAZ signaling [66]. Clinically, this chronic endothelial activation drives aberrant immune cell adhesion and disrupts vascular barrier integrity in human pulmonary fibrosis [67]. Trapped in these activated, anatomically misplaced, or remodeling-associated states, endothelial cells fail to recover a quiescent phenotype, thereby impairing the reconstruction of a functional gas-exchange interface.

### 5.3. Vascular Niche: Regenerative Support Versus Profibrotic Organizer

During distal lung repair, the pulmonary capillary endothelium operates as a critical signaling niche, providing angiocrine cues that instruct epithelial regeneration. For example, in pneumonectomy models, endothelial VEGFR2 and FGFR1 activation sustains alveolar regeneration by maintaining MMP14-dependent epithelial growth signals [68]. Furthermore, angiocrine sphingosine-1-phosphate directly engages AT2 cells via the S1PR2–YAP axis, illustrating how vascular-derived signals actively orchestrate the rebuilding of the alveolar–capillary interface [69].

However, recurrent lung injury subverts this regenerative capacity, driving capillary endothelial cells and perivascular macrophages to form a maladaptive, profibrotic niche. This pathogenic transition is defined by reduced endothelial CXCR7 signaling, concurrent with endothelial *Jag1* induction and subsequent fibroblast Notch activation. The causal role of these altered angiocrine signals is evident, as targeted restoration of CXCR7 activity or suppression of *Jag1* successfully reinstates alveolar repair and attenuates fibrosis [70].

Alveolar–capillary non-resolution reflects a breakdown in this angiocrine coordination. When the vascular niche ceases to instruct epithelial repair and stromal restraint, aberrant endothelial–immune–fibroblast communications actively organize and stabilize the remodeling lesion, effectively transforming the perfused gas-exchange interface into a persistent scar architecture.

### 5.4. Endothelial Reset Failure

While transient endothelial activation, characterized by increased permeability and immune cell trafficking, is essential for the acute injury response, successful tissue resolution requires a subsequent withdrawal toward a quiescent, barrier-stabilizing state. In pulmonary fibrosis, this temporal reset is impaired. The prolonged retention of a leaky, activated vascular bed continuously exposes the tissue to plasma proteins, inflammatory cells, and provisional matrix cues, thereby creating a microenvironment less compatible with resolution [67].

Aging critically disrupts this sequence of temporal reprogramming. Longitudinal analyses of lung injury models demonstrate that while young cohorts successfully reshape pulmonary capillary endothelial subpopulations during the transition from peak fibrosis to regeneration, aging severely stalls this process [66,71]. Injury-activated programs persist rather than recede, trapping the endothelium in a state of prolonged plasticity that fails to rebuild a stable alveolar–capillary interface.

At the molecular level, this failure to restore homeostatic transcriptional control is driven by the impairment of key regulatory pathways. For instance, deficiency of ERG, a master regulator of endothelial homeostasis, precipitates fibrogenic abnormalities and impaired capillary repair that directly mimic aging-associated and human lung fibrosis [72]. Similarly, impaired nitric oxide–soluble guanylate cyclase–cyclic guanosine monophosphate (NO–sGC–cGMP) signaling further links age-related vascular dysfunction to progressive scarring, demonstrating that the failure to deactivate injury-induced communication networks impedes the recovery of a quiescent, alveolar-supportive vascular bed [73].

### 5.5. Aberrant Vascularization Does Not Restore Gas Exchange

Vessel expansion alone does not indicate structural resolution. Alveolar repair requires the appropriate capillary identity, functional barrier integrity, and anatomical alignment with AT1 epithelium. Expansion of COL15A1^+^ endothelial programs illustrates how ectopic vascularization can replace normal alveolar capillary architecture without restoring gas exchange [64,65,74].

The fibrotic lung therefore remains highly vascularized, yet its endothelial programs are anatomically misplaced, persistently activated, and temporally stalled. By adapting to the remodeled stroma instead of reconstituting a thin, apposed epithelial–capillary interface, these aberrant networks actively nourish and maintain a vascularized scar. This failure to reassemble a functional gas-exchange unit defines the structural endpoint of non-resolving repair.

## 6. Cross-Module Integration: How Unresolved Repair Becomes Progressive Fibrosis

While the compartmental failures of epithelial maturation, stromal exit, immune clearance, and vascular reset can be analytically isolated, progressive IPF is not driven by any single aberrant cell state. Rather, the fundamental driver of disease progression is the lesion unit—a spatial domain where these multiple failed repair tasks become locally and inextricably coupled.

### 6.1. From Cell States to Lesion Units

Spatial transcriptomics confirms that IPF tissue is heavily organized into pathogenic and remodeling-associated niches rather than random admixtures of aberrant cells. Within these anatomically defined fields, incomplete epithelial closure, persistent matrix production, unfinished immune clearance, and defective vascular rebuilding effectively lose their separability, demonstrating that non-resolution is highly structured at the tissue level [7,26,27].

**Figure 2:**
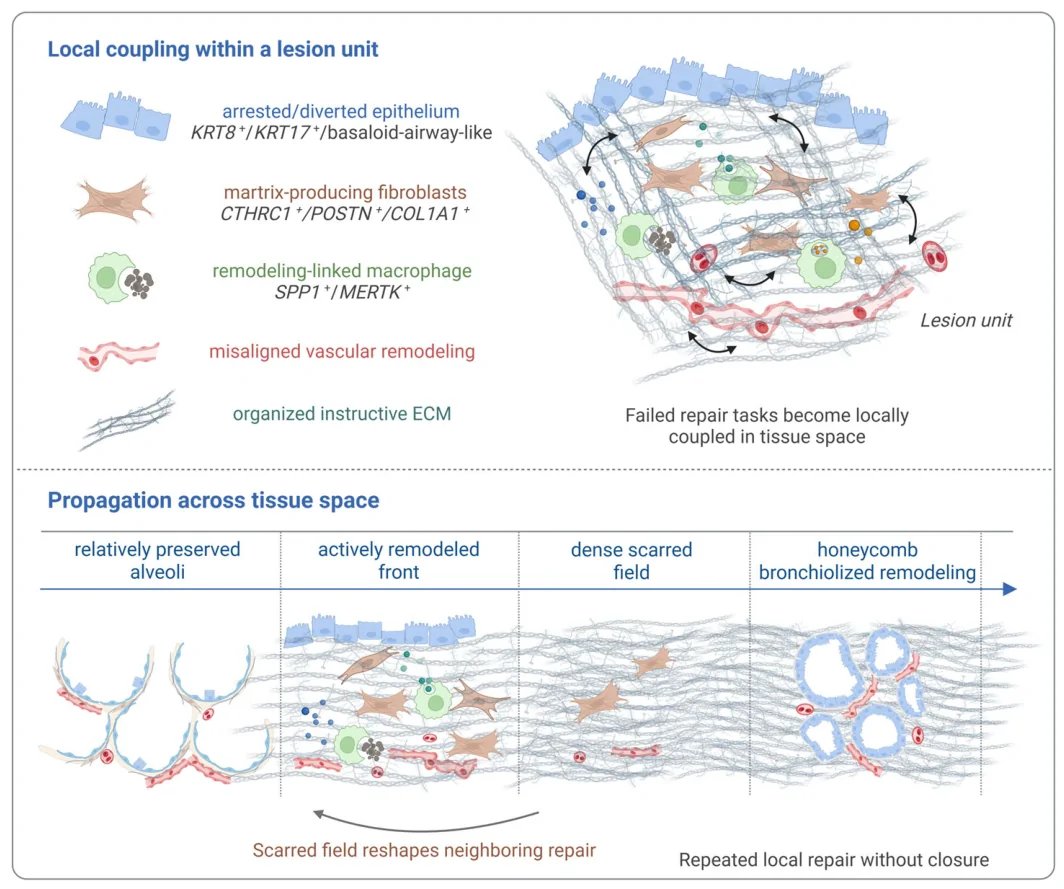
Local coupling and spatial propagation of non-resolving repair in IPF. Module-specific repair failures can become spatially coupled within local fibrotic lesion units. Arrested or diverted epithelial cells, matrix-producing fibroblasts, remodeling-linked macrophages, misaligned vascular elements, and organized instructive ECM occupy the same tissue niche, allowing epithelial maturation failure, stromal exit failure, incomplete immune disengagement, vascular misalignment, and matrix persistence to reinforce one another. Across the lung, these coupled lesion units are embedded within a heterogeneous remodeling landscape in which relatively preserved alveolar units coexist with actively remodeled fronts, dense collagenized scar fields, and honeycomb or bronchiolized cystic remodeling. This arrangement is best interpreted as a spatial continuum of increasing remodeling severity and architectural distortion, not as a strictly linear temporal sequence. Repeated local repair attempts that fail to close may allow scarred matrix fields to reshape adjacent repair niches, ultimately promoting architectural lock-in and limiting return to a functional gas-exchange unit. Abbreviations: ECM, extracellular matrix; IPF, idiopathic pulmonary fibrosis. Created in BioRender. Ge, C. (2026) https://BioRender.com/jby110t.

Crucially, once this local coupling is established, the lesion unit actively dictates its surrounding repair environment. The epithelial edge no longer encounters a normal alveolar template, the stromal compartment is embedded in a highly remodeled matrix, immune cells confront unresolvable cargo, and vascular repair is redirected by distorted interstitial architecture. Spatial niche locking instead represents the tissue-level consequence of multiple failed programs converging into a self-sustaining, progressive lesion. The local coupling of failed repair programs and their spatial propagation are summarized in Figure 2.

### 6.2. Propagation of Non-Resolving Repair

While clinical IPF progression is typically tracked by declining lung function or expanding radiological abnormalities, at the tissue level, it manifests as the spatial propagation of non-resolving repair. Beyond mere collagen accumulation, disease advancement is driven by how a local lesion unit corrupts its surrounding microenvironment, establishing new boundaries of injury and increasing the likelihood that adjacent healthy tissue will be drawn into a similar trajectory of failed repair.

#### 6.2.1. Lesion Boundaries as Active Repair-Remodeling Fronts

Fibroblastic foci epitomize lesion boundaries marked by ongoing repair and remodeling. Within an individual fibroblastic focus, the active fibrotic front lies at the perimeter of the myofibroblast core and extends into adjacent thickened alveolar septa; the core contains nonproliferating myofibroblasts that continue to synthesize collagen [75]. While earlier three-dimensional reconstructions conceptualized these foci as a continuous reticulum [76], advanced micro-computed tomography (micro-CT) and histological analyses reveal them to be highly heterogeneous, discrete sites [77].

At the broader tissue-region scale, fibroblastic foci lie at the boundary between relatively preserved parenchyma and established fibrosis. Spatially resolved transcriptomic and proteomic analyses further delineate this topography, resolving fibroblastic foci and adjacent dense fibrosis as distinct yet coupled compartments in which cellular activity and tissue architecture evolve in tandem [47,78]. Functionally, fibroblastic foci operate as spatially structured interfaces where local injury, stromal expansion, and architectural distortion converge, actively driving the destruction of adjacent alveolar septa and disrupting local microvasculature [79]. Activity at the peripheral fibrotic front consequently alters the mechanical and biochemical conditions under which subsequent local repair occurs.

#### 6.2.2. Dense Fibrosis as a Field That Alters Neighboring Repair

Dense fibrosis comprises mature scar tissue that continues to influence neighboring repair [47]. This scarred matrix profoundly alters local stiffness, basement membrane continuity, and interstitial geometry. Neighboring alveolar regions are forced to initiate repair adjacent to a tissue environment that already harbors the mechanical and biochemical memory of failed resolution.

This “field effect” provides a tissue-level mechanism for the spatial and temporal heterogeneity characteristic of the usual interstitial pneumonia (UIP) pattern, explaining how fibroblastic foci, dense scars, and relatively preserved regions anatomically coexist [27,47,79,80]. Progression, therefore, is not a uniform radial expansion from a single center, but a discontinuous cycle: localized repair failures leave behind distorted fields, which in turn corrupt the repair conditions of bordering preserved lung tissue, continuously recruiting new regions into the fibrotic cascade.

### 6.3. Architectural Lock-In

As these non-resolving repair units multiply, the lung progressively loses the fundamental spatial template required for alveolar reconstruction. Each active remodeling front generates a scarred field, which subsequently compromises adjacent repair attempts—creating a vicious cycle where each localized failure increases the probability of the next. Ultimately, disease propagation evolves into a profound architectural deficit. Because repeated lesions irrevocably distort the structural scaffold, therapeutic interventions that only suppress matrix production or induce partial collagen clearance may reduce the absolute fibrotic burden, but they critically fail to reorganize a functional gas-exchange interface.

#### 6.3.1. Fibrotic ECM as an Organized and Instructive Architecture

Rather than a diffuse collagenous mass, the ECM in IPF possesses a highly reproducible internal organization [75]. Proteomic profiling of microdissected lesions reveals that fibroblastic foci, mature scars, and adjacent alveolar regions maintain distinct protein compositions, establishing matrix architecture as a highly localized property [78].

Crucially, this spatially organized ECM exerts an active, instructive role. Decellularized IPF matrix directly transmits fibrogenic signals to resident cells, driving a self-sustaining feedback loop in normal fibroblasts [81] and modulating aberrant KRT5^+^ basal cell behaviors within the fibrotic niche [82]. Consequently, any cellular attempt at physiological repair must navigate an entrenched ECM landscape that continuously broadcasts pathological structural and biochemical cues.

#### 6.3.2. Matrix Removal Is Not Equivalent to Repair

Advanced architectural distortion, most notably honeycomb remodeling, illustrates why matrix clearance alone is insufficient for functional restoration. In these severely remodeled distal regions, the alveolar scaffold is severely disrupted, replaced by cystic structures lined by a mucociliary pseudostratified epithelium [83]. Concurrently, the vascular bed undergoes ectopic, airway-like expansion, leaving vessels physically present but functionally uncoupled from the remodeled epithelium [64,65,74].

Within these geometrically distorted confines, degrading scar tissue may reduce local collagen volume. Tissue repair would necessitate the de novo reconstruction of alveolar wall geometry, specialized epithelial identity, and apposed capillary placement. Thus, “architectural lock-in” dictates a strict hierarchy in therapeutic outcomes: while suppressing matrix production is a necessary prerequisite for halting progression, achieving functional respiratory repair demands the structural reassembly of a previously destroyed physiological scaffold.

### 6.4. Resolving Injury Versus Human IPF: Why Repair Fails to Close

Although resolving experimental injury and human IPF share similar transcriptional injury-response programs, their capacity for tissue closure differs. The emergence of transitional or activated cell states alone does not denote pathology; their biological significance depends on whether they regress toward homeostasis or persist within progressive remodeling. For instance, murine *Krt8*^+^ transitional alveolar epithelial cells successfully mediate regeneration and subsequently resolve, whereas analogous intermediates in human IPF persist under chronic stress and aberrantly evolve into basaloid states [11,15].

#### 6.4.1. Injury Tempo and the Synchrony of Repair

This functional divergence is primarily rooted in injury tempo and synchrony. Experimental models typically utilize a singular, defined insult, generating a synchronized wave of inflammation, fibrosis, and resolution; this temporal alignment allows activated stromal compartments (such as *Cthrc1*^+^ myofibroblasts) to reversibly switch back to lipofibroblast-like states [42].

Conversely, IPF is thought to involve repeated, spatially asynchronous micro-injuries, meaning the lung never enters a unified repair stage. Consequently, in human IPF, “time is folded into space”: relatively preserved parenchyma, fibroblastic foci, and dense scars anatomically coexist [27,47]. Because lesions are organized along gradients of remodeling severity rather than a synchronized timeline, an isolated cell state in IPF is inextricably trapped within a distorted niche, rendering its biological trajectory different from an identical cell state in a resolving murine wound.

#### 6.4.2. Aging and Reduced Reset Capacity

Furthermore, advanced age critically depletes the intrinsic “reset capacity” required for physiological resolution across all tissue axes. In the alveolar epithelium, telomere dysfunction drives senescence and propagates profibrotic signals [24,25]. Simultaneously, stromal reset is obstructed by age-related Nox4–Nrf2 redox imbalances, which promote the accumulation of apoptosis-resistant myofibroblasts and impair dedifferentiation [84,85].

This loss of reversibility extends seamlessly into the immune and vascular systems: hematopoietic aging skews monocyte-derived macrophages away from resolving efferocytosis toward profibrotic remodeling [52,54,86], while aged pulmonary capillaries suffer from rarefaction and impaired endothelial nitric oxide synthase (eNOS)-dependent reprogramming [71,73]. Thus, aging amplifies the initial injury response while narrowing the pathways by which cellular programs return to baseline homeostasis.

Taken together, progressive pulmonary fibrosis transcends isolated cellular dysfunction to operate as a self-propagating architectural disorder. As compartmental failures converge into integrated lesion units, they generate a pathologically altered field that dictates the biological trajectory of adjacent tissues. Compounded by asynchronous micro-injuries and an age-related decline in cellular reset capacity, these active remodeling fronts progressively dismantle the spatial alveolar–capillary template. The failure of tissue closure in human IPF therefore represents a structural impasse. Regenerative cellular programs remain active, yet they are trapped within a distorted physical and biochemical scaffold that precludes physiological reconstruction. Recognizing this tissue-level integration redefines the therapeutic challenge, shifting the paradigm from suppressing aberrant cell states to overcoming the anatomical constraints that prevent true tissue resolution.

## 7. Mapping Resolution Capacity Beyond Cell States

Following the realization that non-resolving repair is spatially locked at the tissue level, the next functional imperative is to evaluate whether a microenvironmental domain retains a viable route to tissue closure. The biological trajectory of an aberrant cellular state cannot be inferred from its transcriptional identity alone; rather, its capacity for reversibility is dictated by the architectural constraints of its specific lesion environment. This dependency shifts the focus of tissue atlasing toward resolution capacity—the residual potential of a fibrotic region to transition from chronic remodeling back to coordinated repair.

**Figure 3:**
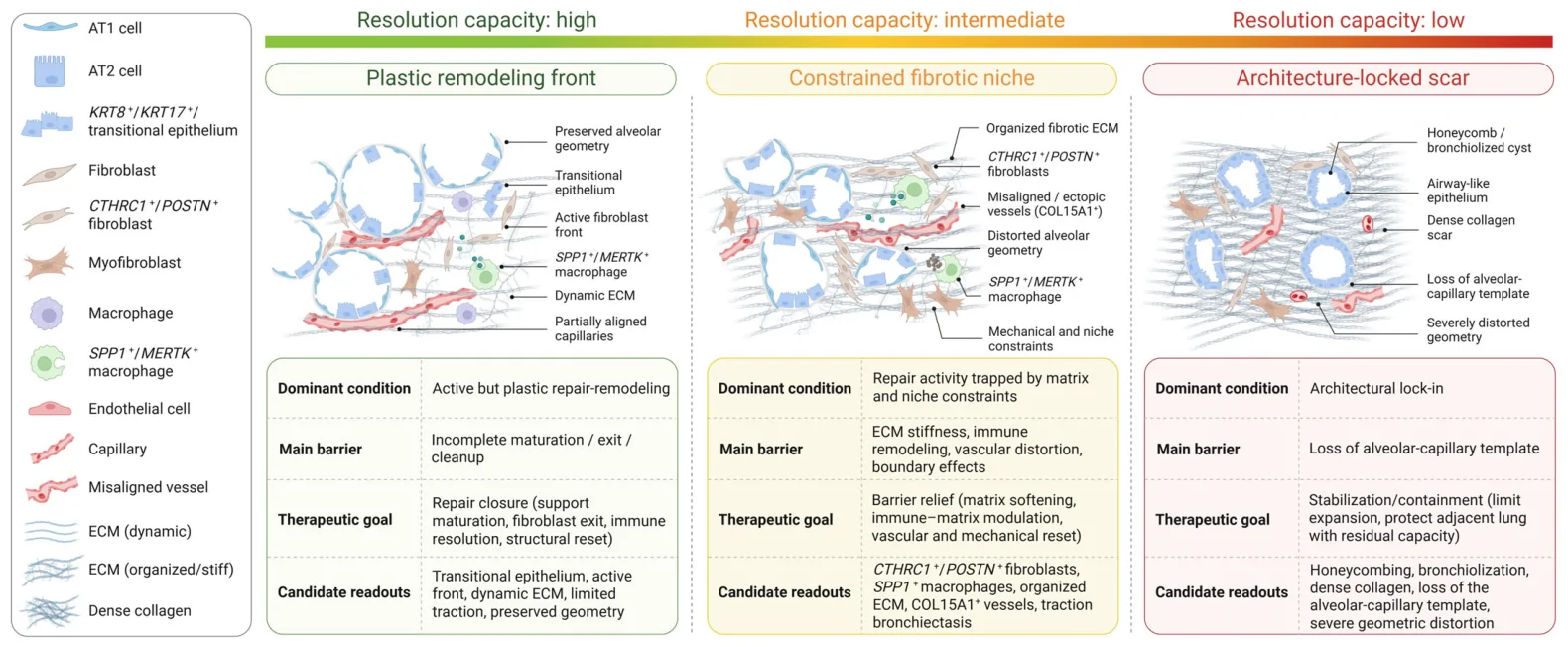
Resolution-capacity states and lesion-state–matched intervention in IPF. Fibrotic lesions in IPF can be conceptualized as regions with different degrees of residual resolution capacity rather than as uniform scar tissue. A plastic remodeling front contains active injury-repair programs, dynamic ECM remodeling, partially preserved alveolar geometry and cell states that may still retain maturation, withdrawal or reset potential. In this context, the therapeutic goal is repair closure, including epithelial maturation, fibroblast exit, immune resolution and vascular reset. A constrained fibrotic niche contains persistent epithelial, stromal, immune and vascular remodeling programs embedded within organized matrix, distorted vascular/airway relationships and local mechanical constraints. Here, therapy may need to relieve coupled barriers, including matrix stiffness, profibrotic immune remodeling, vascular misplacement and niche-dependent reinforcement. An architecture-locked scar is characterized by dense collagen deposition, honeycomb or bronchiolized cystic remodeling, airway-like epithelium, loss of the alveolar–capillary template, impaired epithelial–capillary apposition and distorted alveolar–capillary geometry. In such regions, local reversal may be limited, and therapeutic goals may shift toward stabilization, containment and preservation of adjacent lung. These states are not strict temporal stages; they may coexist within the same IPF lung and define region-specific opportunities and limits for repair-oriented intervention. Abbreviations: ECM, extracellular matrix; IPF, idiopathic pulmonary fibrosis. Created in BioRender. Ge, C. (2026) https://BioRender.com/wnpce2u.

Fibrotic lesions should therefore be stratified along a repair-closure axis spanning highly plastic remodeling fronts, constrained niches, and architecture-locked scars. To transition IPF atlasing from description to therapeutic decision-making, this local reversibility must be systematically mapped across three integrative dimensions: protein and ECM compartmentalization, three-dimensional lesion geometry, and quantitative regional imaging. These resolution-capacity states and their corresponding intervention logic are summarized in Figure 3.

### 7.1. Protein and ECM Environment: Biochemical Permissiveness for Repair

Because tissue resolution largely depends on extracellular matrix (ECM) remodeling and proteolytic turnover, transcriptomic maps alone are insufficient to gauge true repair capacity. Critical determinants of fibrotic progression, such as secreted mediators, matrix-bound growth factors, and localized ECM compartmentalization, operate at the protein level. Therefore, assessing whether a microenvironment remains biochemically permissive for tissue reconstruction requires shifting the focus from cellular transcriptomes to spatially resolved protein dynamics.

Advanced spatial molecular imaging, including multiplexed protein profiling, matrix-assisted laser desorption/ionization (MALDI) mass spectrometry, and spatial metabolomics, now enables the interrogation of IPF lesions as defined biochemical coordinates. Building upon the previously established structural heterogeneity of the fibrotic matrix, these technologies can distinctly segregate an active remodeling front (enriched for dynamic matrix turnover and immune-remodeling signals) from a mature scar (dominated by cross-linked, persistent matrices that physically impede epithelial or vascular reconstruction). By comparing these spatial readouts between resolving murine injuries and human fibrotic persistence, it becomes possible to pinpoint disease-relevant shifts, such as altered glycogen metabolism, that are not readily captured by standard cell-state analyses [75,78,87,88,89].

A resolution-oriented molecular map must transcend simple collagen quantification. It must systematically delineate whether basement membrane integrity is preserved or fragmented, whether matrix-bound signals actively sustain TGF-β and integrin pathways, and whether proteolytic patterns indicate dynamic tissue plasticity rather than a terminally fixed scar. This comprehensive biochemical stratification provides the necessary framework to separate lesions that retain latent repair potential from those whose protein and ECM landscape constitutes an insurmountable barrier to closure.

### 7.2. Three-Dimensional Lesion Geometry and Anatomical Constraints

Assessing resolution capacity requires transitioning from two-dimensional histological perspectives to volumetric lesion geometry. Fibrotic niches operate as three-dimensional structures that physically constrain tissue repair. Micro-CT and advanced histological reconstructions reveal that fibroblastic foci possess complex, branching topologies extending well beyond single cross-sections [77]. The topological features of a lesion, including its size, continuity, and boundary sharpness, may constrain the physical degrees of freedom available for cellular reorganization. A discrete active front adjacent to preserved alveoli retains greater morphogenetic flexibility than a continuously interconnected remodeling field embedded within dense scar tissue.

This geometric constraint scales from microscopic niches to macroscopic architectural deformation. The profusion of fibroblastic foci correlates with high-resolution CT abnormalities, particularly traction bronchiectasis severity, demonstrating how localized active remodeling drives large-scale tissue collapse [90]. Equivalent cellular programs therefore exhibit variable reversibility based on their geometric context. An active repair niche situated within preserved local geometry maintains biological plasticity, whereas identical cellular activity coexisting with severe airway traction, vascular displacement, or alveolar collapse is mechanically locked.

Macroscopic anatomical boundaries impose additional biomechanical constraints on tissue resolution. The characteristic subpleural and peripheral predominance of IPF indicates that pleural proximity influences the stabilization of non-resolving repair. Experimental and human studies show that pleural mesothelial cells traffic into the lung parenchyma and engage in pleural–stromal communication to drive fibrogenesis [91,92,93]. Beyond their cellular contributions, these boundary-positioned lesions are subjected to distinct mechanical anchoring, altered matrix organization, and unique interstitial tension. Such peripheral biomechanics accelerate the stabilization of remodeling fields, extinguishing spatial plasticity and securing the lesion against the pleural interface, which limits residual routes for alveolar reconstruction.

### 7.3. Quantitative Imaging and AI-Assisted Integration of Resolution-Capacity States

Integrative mapping connects biochemical and geometric data to whole-lung disease organization. Assessing resolution capacity requires this multiscale approach, as isolated metrics, such as collagen area, Ashcroft score, alpha-smooth muscle actin (α-SMA) expression, forced vital capacity (FVC) decline, and visual computed tomography (CT) fibrosis extent, represent distinct biological levels. For example, local fibrotic markers may decrease without architectural restoration, and stable radiological fibrosis does not guarantee epithelial-capillary reconstruction.

Quantitative high-resolution computed tomography (qHRCT) and artificial intelligence (AI) provide scalable tools to bridge these spatial levels. qHRCT fibrosis scoring offers reproducible measurements of fibrotic burden and predicts short-term clinical progression [94,95]. Functional imaging-based quantitative CT has also been reported to describe IPF progression more effectively than forced vital capacity in selected settings [96]. Deep learning models further extract latent structural information by converting complex CT patterns into quantitative variables, such as specific fibrosis, vessel, and airway volumes [97]. These imaging-derived metrics predict IPF progression and mortality independent of visual assessments [98]. Linking this macroscopic latent information to spatial molecular features allows non-invasive imaging to reflect underlying lesion states.

Combining spatial multi-omics, three-dimensional pathology, and quantitative imaging yields an integrated resolution-capacity map. This framework classifies fibrotic regions into biologically meaningful states based on their residual repair potential. A plastic remodeling front features active injury responses and dynamic ECM within preserved local geometry, indicating that targeted interventions might still achieve tissue closure. A constrained remodeling lesion pairs persistent cell-state activity with organized matrix or vascular distortion, requiring multiple barriers to be addressed simultaneously. An architecture-locked scar is characterized by dense matrix, honeycomb remodeling, and epithelial-capillary misalignment. In these locked regions, structural routes for local reversal are lost, shifting the therapeutic focus toward stabilization and preserving adjacent tissue.

Integrating these biological and radiological dimensions transitions IPF atlasing from cellular description to clinical decision-making. A catalog of abnormal cell states holds limited translational value unless it distinguishes active fibrotic remodeling from constrained repair and advanced architectural lock-in. Assigning lesion regions to specific resolution-capacity states provides a practical bridge to therapy, allowing treatments to be evaluated based on the specific biological and structural barriers present in each local tissue context.

## 8. Therapeutic Reframing: From Disease Slowing to Lesion-State Intervention

Pirfenidone and nintedanib, together with the recently approved preferential phosphodiesterase 4B inhibitor nerandomilast, demonstrate that the clinical course of IPF is pharmacologically modifiable [99,100,101]. Pirfenidone attenuates TGF-β-associated fibroblast activation and matrix synthesis; nintedanib inhibits PDGFR, FGFR, and VEGFR signaling, thereby limiting fibroblast proliferation and extracellular matrix deposition [102,103,104]. Nerandomilast enhances cAMP signaling through preferential PDE4B inhibition and has been shown to reduce myofibroblast contractility and improve endothelial barrier function [105].

These agents therefore attenuate selected active components of non-resolving repair. All three slowed FVC decline, but lesion-level repair closure and restoration of alveolar–capillary architecture were not assessed. In established lesions, persistent epithelial, stromal, immune, and vascular abnormalities become coupled to matrix stiffening and geometric distortion. Reducing ongoing fibrotic activity may slow disease progression while these interdependent constraints on repair closure persist. Whole-lung functional benefit can therefore coexist with regional non-resolution. A patient experiencing slower functional decline may simultaneously harbor active remodeling fronts, constrained niches, and terminally locked scars. As a whole-lung measure, FVC cannot distinguish these lesion states or identify which repair barrier has changed. The translational challenge is to match therapeutic interventions to the specific resolution capacity of these diverse regions.

### 8.1. Lesion-State–Matched Therapeutic Goals

In highly plastic remodeling fronts, local repair tasks may still be re-engaged. Preclinical studies identify several routes for promoting repair completion. TRβ activation promotes AT2-to-AT1 differentiation and lung epithelial-specific Wnt activation supports alveolar regeneration [106,107]. Metformin accelerates fibrosis resolution by directing myofibroblasts toward a lipogenic state [41]. Extracellular-vesicle–mediated signals have also supported alveolar progenitor renewal and recovery of alveolar structure in experimental fibrosis [108,109]. Conversely, in constrained fibrotic niches, the therapeutic objective shifts to barrier relief. Clinical trial experiences, including the failure of agents targeting LOXL2 (simtuzumab) [110], integrin [111], connective tissue growth factor (pamrevlumab) [112], and autotaxin (ziritaxestat) [113], highlight the difficulty of this approach. Targeting a single profibrotic pathway often yields limited clinical benefit if the dominant microenvironmental constraint remains unaddressed or if the targeted region lacks residual resolution capacity. Finally, for architecture-locked scars dominated by honeycomb remodeling, restoring the alveolar–capillary template is improbable [83,114]. Therapeutic goals for these advanced lesions must pivot toward structural stabilization and the preservation of bordering healthy tissue.

### 8.2. Coupled Barriers, Therapeutic Timing, and Combination Strategies

This regional heterogeneity redefines the rationale for combination therapy. A systemic drug enters a landscape where its target pathway may act as a primary driver in one niche but a secondary consequence in another. Combination strategies should therefore be guided by local dependencies rather than simply combining multiple antifibrotic agents. For instance, promoting epithelial maturation may prove ineffective if the surrounding matrix remains mechanically restrictive, and fibroblast targeting may be unstable if macrophage-driven activation cues persist. In these settings, therapeutic timing depends on identifying which specific structural or biochemical barrier must be alleviated first to allow subsequent repair programs to proceed.

### 8.3. Resolution-Oriented Endpoints and Trial Design

A lesion-state therapeutic framework requires endpoints that link patient benefit with regional tissue behavior. FVC, mortality, acute exacerbation, symptoms and quality of life remain indispensable because they capture outcomes that matter to patients. Their biological resolution is limited. They cannot identify which repair barrier changed, which lesion regions responded or whether a treatment produced marker suppression without structural improvement. Recent endpoint discussions in IPF have therefore called for patient-meaningful endpoints with improved reliability and biological interpretability, including composite outcomes, imaging, physiologic measures and biomarkers [115,116].

Regional structural endpoints offer critical spatial context. qHRCT and AI-assisted imaging, including quantitative lung fibrosis (QLF) scoring, enable the longitudinal tracking of regional disease trajectories, directly evaluating whether a therapy modifies the structural evolution of the targeted lesion landscape. QLF has been evaluated as an efficacy endpoint in recent phase II trials, where a clinically meaningful threshold has been estimated for fibrotic burden change [117,118]. Integrating such imaging modalities is essential for differentiating among continued lesion expansion, architectural stabilization, and bona fide structural regression.

To mechanistically underpin these structural and clinical signals, lesion-level molecular endpoints are needed. Historically, early-phase IPF trials have relied on bulk or systemic biomarkers to confirm target engagement [119]. However, under the non-resolving repair paradigm, these molecular readouts must be spatially contextualized. Emerging high-throughput technologies, such as spatial transcriptomics, ECM registration, region-resolved proteomics, and multiplexed immune decoding, allow specific pathological niches and cellular programs to be anatomically mapped [7,75,120]. Lesion-level molecular endpoints should identify where a molecular change occurs, whether the intended target is engaged within that tissue context, and whether the change aligns with local structural stabilization or repair-compatible organization.

**Figure 4:**
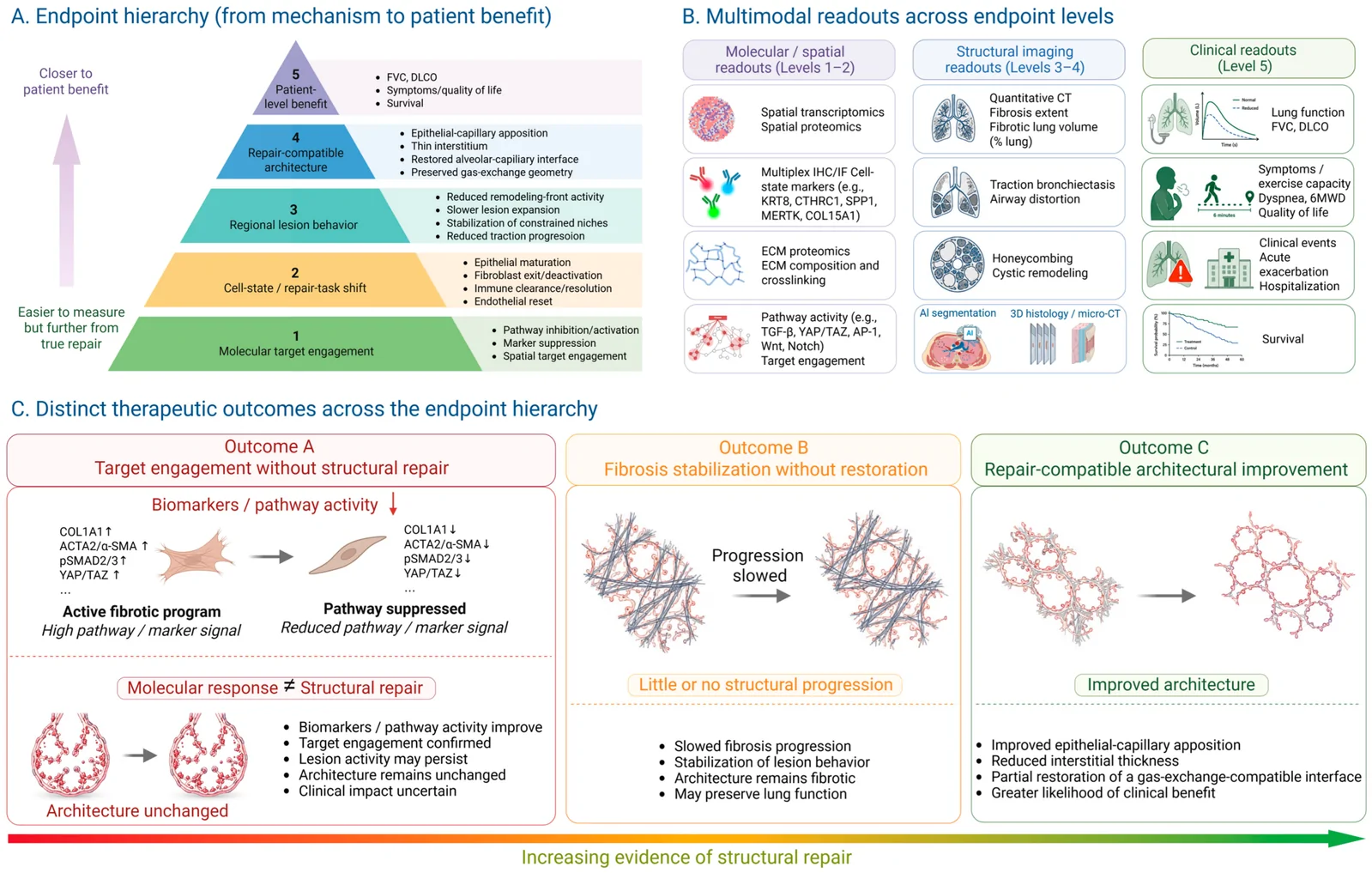
Endpoint hierarchy for repair-oriented therapy in IPF. Repair-oriented therapy in IPF requires endpoints that distinguish molecular target engagement, cell-state or repair-task shifts, regional lesion behavior, repair-compatible architecture and patient-level benefit. Panel A organizes these outcomes into a hierarchy, from pathway inhibition or spatial target engagement at Level 1, to epithelial maturation, fibroblast exit/deactivation, immune cleanup/resolution and endothelial reset at Level 2, to changes in regional lesion behavior at Level 3, repair-compatible tissue architecture at Level 4 and clinical benefit at Level 5. Panel B illustrates multimodal readouts aligned to these levels. Molecular and spatial readouts, including spatial transcriptomics, spatial proteomics, multiplex imaging, ECM proteomics and pathway-activity measures, can assess Levels 1–2. Structural and architectural readouts, including quantitative CT, fibrosis extent, traction bronchiectasis, airway distortion, honeycombing, cystic remodeling, AI-based segmentation, 3D histology and micro-CT, can assess Levels 3–4. Clinical readouts, including FVC, DLCO, dyspnea, 6MWD, quality of life, acute exacerbation, hospitalization and survival, assess Level 5. Panel C emphasizes that therapeutic signals should not be conflated. Target engagement without structural repair indicates biomarker or pathway suppression while fibrotic architecture remains unchanged. Fibrosis stabilization indicates slowed lesion expansion or reduced progression while architecture remains fibrotic. Repair-compatible architectural improvement requires movement toward improved epithelial–capillary apposition, reduced interstitial thickness and partial restoration of a gas-exchange-compatible interface. True repair-oriented assessment should therefore evaluate therapies across the hierarchy rather than equating marker suppression or disease stabilization with architectural repair. Abbreviations: 3D, three-dimensional; 6MWD, six-minute walk distance; AI, artificial intelligence; CT, computed tomography; DLCO, diffusing capacity of the lung for carbon monoxide; ECM, extracellular matrix; FVC, forced vital capacity; IPF, idiopathic pulmonary fibrosis; micro-CT, micro-computed tomography. Created in BioRender. Ge, C. (2026) https://BioRender.com/ektxm16.

Functional reconstruction remains the highest benchmark, since true resolution would require restoration of the distal gas-exchange unit. This outcome is rarely measured directly in human IPF trials. A practical near-term endpoint hierarchy should therefore connect patient-level benefit with regional structural behavior, lesion-level molecular change and, when feasible, evidence that gas-exchange organization is preserved or partially restored. This hierarchy would help distinguish outcomes that are often conflated: slowed functional decline, stabilization of fibrotic architecture, movement of a defined repair barrier and restoration of repair-compatible tissue organization. This repair-oriented endpoint hierarchy is summarized in Figure 4.

A lesion-state endpoint framework also changes how negative trials should be interpreted. A failed study may indicate that the target is insufficient to modify disease course, but it may also reflect lesion-state mismatch, treatment outside the responsive window, insufficient enrichment, endpoint insensitivity, or persistence of coupled barriers after one pathway has shifted. Future IPF trials should therefore specify the lesion composition or active-barrier profile they aim to modify, define the expected regional response, and test whether that response translates into structural and clinical benefit. Finally, therapy becomes the practical extension of resolution-capacity mapping: identifying which regions retain therapeutic opportunity, defining what prevents repair from proceeding there, and matching both treatment and endpoints to that regional barrier.

## 9. Conclusions and Perspectives

Single-cell and spatial omics have reframed IPF as a disorder in which repair programs persist after the conditions required for their completion have deteriorated. Far from a passive accumulation of inert scar tissue, the fibrotic lung operates as an actively remodeling organ where continuous cellular repair attempts remain uncoupled from structural restoration. Isolated disease-associated cell states lack fixed biological meaning. A transcriptional profile that mediates physiological healing in a resolving wound drives progressive fibrosis when trapped within a lesion environment that restricts cellular maturation, phenotype exit, and niche resetting. The lesion unit serves as the convergence point for these interdependent failures, where localized arrests in tissue regeneration mutually reinforce one another, corrupt adjacent architecture, and precipitate subsequent cycles of incomplete repair.

Advancing pulmonary fibrosis research requires shifting the focus from cataloging aberrant transcriptomes toward prospectively mapping resolution capacity. Comprehensive tissue atlasing must integrate spatial omics, region-resolved proteomics, three-dimensional pathology, and quantitative imaging to evaluate the residual reversibility of specific microenvironments. This multidimensional approach enables the stratification of fibrotic tissue into distinct biological categories: plastic remodeling fronts retaining regenerative potential, constrained niches hindered by specific biochemical or geometric barriers, and architecture-locked scars harboring severe structural deformation with limited reversibility.

Therapeutic development must align with this lesion-centered paradigm. While existing antifibrotic agents provide crucial disease-slowing benefits, advancing toward repair-oriented therapy requires precise identification of the local barriers preventing tissue closure. Depending on the spatial context, interventions must be tailored to alleviate specific microenvironmental constraints or to stabilize advanced regions where structural reversal is unachievable. Clinical trial endpoints must parallel this precision, distinguishing localized marker suppression from macroscopic fibrosis stabilization and physiological tissue reconstruction. Recognizing that established idiopathic pulmonary fibrosis contains zones of severe architectural distortion with limited reversibility, the non-resolving repair framework provides a measurable basis for evaluating disease progression. By delineating which regions retain resolution capacity and characterizing their dominant barriers, this approach offers a rational foundation for guiding fibrotic tissue back toward organized functional repair.

## Data Availability

No new data were created or analyzed in this study. Data sharing is not applicable to this article.

## Abbreviations

3D, three-dimensional; 6MWD, six-minute walk distance; α-SMA, alpha-smooth muscle actin; aCap, aerocyte capillary endothelial cell; ADI, alveolar differentiation intermediate; AI, artificial intelligence; AT1, alveolar type 1 epithelial cell; AT2, alveolar type 2 epithelial cell; cGMP, cyclic guanosine monophosphate; CT, computed tomography; DATP, damage-associated transient progenitor; DLCO, diffusing capacity of the lung for carbon monoxide; ECM, extracellular matrix; ER, endoplasmic reticulum; FVC, forced vital capacity; gCap, general capillary endothelial cell; IL-1β, interleukin-1 beta; IPF, idiopathic pulmonary fibrosis; MALDI, matrix-assisted laser desorption/ionization; M-CSF, macrophage colony-stimulating factor; M-CSFR, macrophage colony-stimulating factor receptor; micro-CT, micro-computed tomography; Mo-AM, monocyte-derived alveolar macrophage; NO, nitric oxide; NSIP, nonspecific interstitial pneumonia; PATS, pre-alveolar type 1 transitional cell state; qHRCT, quantitative high-resolution computed tomography; QLF, quantitative lung fibrosis; sGC, soluble guanylate cyclase; SPM, specialized pro-resolving mediator; TGF-β, transforming growth factor beta; TRβ, thyroid hormone receptor beta; UIP, usual interstitial pneumonia; YAP/TAZ, yes-associated protein/transcriptional coactivator with PDZ-binding motif.
